# A Density Functional Theory-Based Particle Swarm Optimization Investigation of Metal Sulfide Phases for Ni-Based Catalysts

**DOI:** 10.3390/nano15110788

**Published:** 2025-05-23

**Authors:** Houyu Zhu, Xiaohan Li, Xiaoxin Zhang, Yucheng Fan, Xin Wang, Dongyuan Liu, Zhennan Liu, Xiaoxiao Gong, Wenyue Guo, Hao Ren

**Affiliations:** 1 Shandong Key Laboratory of Intelligent Energy Materials, School of Materials Science and Engineering, China University of Petroleum (East China), Qingdao 266580, China; 2 State Key Laboratory of Petroleum Molecular & Process Engineering, SINOPEC Research Institute of Petroleum Processing Co., Ltd., Beijing 100083, China

**Keywords:** particle swarm optimization, density functional theory, sulfide phase structure, nickel, formation mechanism

## Abstract

Nickel (Ni) catalysts have numerous applications in the chemical industry, but they are susceptible to sulfurization, with their sulfurized structures and underlying formation mechanisms remaining unclear. Herein, density functional theory (DFT) combined with the particle swarm optimization (PSO) algorithm is employed to investigate the low-energy structures and formation mechanisms of sulfide phases on Ni(111) surfaces, especially under high-sulfur-coverage conditions where traditional DFT calculations fail to reach convergence. Using ($\sqrt{3}\times \sqrt{3}$ ) Ni(111) slab models, we identify a sulfurization limit, finding that each pair of deposited sulfur atoms can sulfurize one layer of three Ni atoms at most (Ni:S = 3:2), with additional sulfur atoms penetrating deeper layers until saturation. Under typical reactive adsorption desulfurization conditions, the ab initio thermodynamics analysis indicates that Ni_3_S_2_ is the most stable sulfide phase, consistent with sulfur K-edge XANES data. Unsaturated phases, including Ni_3_S, Ni_2_S, and Ni_9_S_5_, represent intermediate states towards saturation, potentially explaining the diverse Ni sulfide compositions observed in experiments.

## 1. Introduction

Nickel (Ni) catalysts play a pivotal role in the chemical industry, particularly in processes such as desulfurization [1,2,3,4,5,6,7], (de)hydrogenation [8,9,10,11,12,13,14], steam reforming [15,16], and CO/CO_2_ methanation [17,18,19,20,21], due to their high activity, selectivity, and cost-effectiveness. These catalysts are indispensable for refining crude oil and upgrading biomass feedstocks into valuable chemicals and fuels [1,2,3,4,5,6,7,8,9,10,11,12,13,14,15,16,17,18,19,20,21]. However, in real-world chemical reaction environments, the presence of sulfur, inherent in many feedstocks like petroleum fractions, poses a significant challenge. Sulfur can interact with nickel, leading to its sulfurization, which alters the structure and properties of Ni-based catalysts and also impacts their performance and stability over time. Despite the extensive use of Ni-based catalysts, the current understanding of the sulfurization state of active Ni species remains limited, and the mechanisms governing this transformation are not fully elucidated, highlighting the need for more in-depth studies to optimize these catalytic systems.

Generally, research into the specific structural characteristics of Ni sulfide phases should take precedence over studies on their catalytic performance. For instance, some studies suggest that the formation of Ni sulfide phases enhances the hydrodesulfurization (HDS) activity of MoS_2_ catalysts [22,23], while others explicitly state that Ni sulfide phases do not possess HDS activity [24]. To resolve this contradiction, it is essential to first determine the exact structure of Ni sulfide phases under the relevant conditions. Only then can we clarify the true level of their reactivity, thereby providing a more accurate understanding of their catalytic behavior. To be more specific, the Ni/ZnO catalyst used in reactive adsorption desulfurization (RADS) is taken as an example for the illustration of nickel sulfide structures. Huang et al. [25,26] carried out a sulfur K-edge X-ray absorption near-edge structure (XANES) measurement to investigate the RADS process of oil products over Ni/ZnO and found that the organic sulfur compounds are decomposed on the surface Ni of Ni/ZnO to form Ni_3_S_2_. Song et al. [6] also observed by X-ray diffraction (XRD) experiments that Ni is sulfurized to the Ni_3_S_2_ phase in RADS reactions. However, Kong et al. [27] demonstrated, by XRD, that under RADS conditions, the newly formed sulfide phase of Ni is NiS. Additionally, Aray et al. [28] performed a first-principles study of small nickel sulfide particles, and suggested that at typical working conditions, very small particles of non-supported nickel sulfide should be mainly present as Ni_3_S_4_ clusters. The experimental and theoretical studies have not reached a unified and consistent conclusion for the various structures of Ni sulfides with different ratios of Ni to S. Therefore, in most literature reports, researchers typically use Ni_x_S_y_ or NiS_x_ to denote the sulfides of Ni [29,30,31,32,33,34], meaning that there is still a relatively limited understanding of the structure of Ni sulfides under a certain reaction conditions.

To tackle the above issues, we performed a comprehensive study on the sulfide-phase structures of Ni(111) surfaces and the corresponding formation mechanism. The particle swarm optimization (PSO) method was applied, and the energy landscape was sampled with density functional theory (DFT) calculations. The PSO method can efficiently explore the multidimensional potential energy surfaces of a periodic system and requires only known information of chemical compositions to predict the stable structure. This method is successful in predicting two-/three-dimensional crystal structures for various systems ranging from elements to ternary compounds [35,36,37,38,39,40,41,42,43,44,45]. Ni(111) is chosen here because it has been identified as the most thermodynamically stable surface of bulk Ni in XRD experiments [45], and can be considered the most exposed surface of large Ni nanoparticles. The main focus is to preliminarily clarify the sulfurized structures formed on the Ni(111) surface and the effect of the reaction environment on the Ni sulfide phase, which are essentially different from classic S adsorption behaviors based on Ni slab models [7,46,47,48].

## 2. Computational Details

An unbiased swarm-intelligence structural method based on the Particle Swarm Optimization (PSO) technique implemented in the Crystal structure AnaLYsis by Particle Swarm Optimization (CALYPSO) code [49,50] was employed to search for low-energy structures of nickel sulfide phases. Four main steps are included for the structure prediction: (i) the generation of random structures constrained within 230 space groups, (ii) local structural optimization, (iii) post-processing for the identification of unique local minima by bond characterization matrix, and (iv) the generation of new structures by PSO for iterations. The PSO algorithm derives its fundamental concept from observations of avian foraging behavior, where bird flocks collectively share information to locate optimal feeding locations, and the behavior of each individual is affected by either the best local or the best global individual to help it fly through the hyperspace. Moreover, an individual can learn from its past experiences to adjust its flying speed and direction, so all the individuals in the swarm can quickly converge to the global position [51]. In this work, a fixed number of atoms are first deposited on a model surface, and then an ensemble of surface structures is constructed without bias. The deposited atoms include the Ni and S atoms, which are randomly attached to each other and the model surface. The surface structures of nickel sulfide phases evolve towards the structures with lower-energy, both locally and globally, through self- and swarm-structure learning (see the detailed protocol in the Supplementary Materials). For all cases, 10 generations of optimization were performed, and each generation contained 30 individuals. Therefore, up to 300 structures were sampled for each sulfurized surface. For simplicity, five structures with the global minimum energies for each sulfurized surface are presented in Figures S1–S4 of the Supplementary Materials, and among them, the structure with the lowest energy is chosen to represent the nickel sulfide surface.

DFT calculations were performed with the Vienna ab initio simulation package (VASP) [52,53] using the projector augmented wave method (PAW) [54,55] to describe the interaction between core and valence electrons. The exchange correlation effect was processed using generalized gradient approximation developed by Perdew–Burke–Ernzerhof (GGA-PBE) [52]. Plane waves were included for the electronic wave functions up to a cutoff energy of 400 eV. The convergence criteria for the energy and force were set at 10^−6^ eV and 0.03 eV/Å, respectively. The lattice constant of bulk Ni was fitted by the Birch-Murnaghan equation of state, based on a 3.524 Å × 3.524 Å × 3.524 Å Ni cubic cell. The corresponding Brillouin zone was sampled with 15 × 15 × 15 *k*-point meshes [56]. The lattice constant of bulk Ni was calculated to be 3.521 Å, in good agreement with the experimental value (3.524 Å) [57]. All calculations were performed with spin polarization. Dipole corrections were tested, and the energetic discrepancy between pre- and post-dipole correction states remains below 0.01 eV (see Table S1 of the Supplementary Materials), demonstrating a negligible dipole-induced effect.

**Figure 1:**
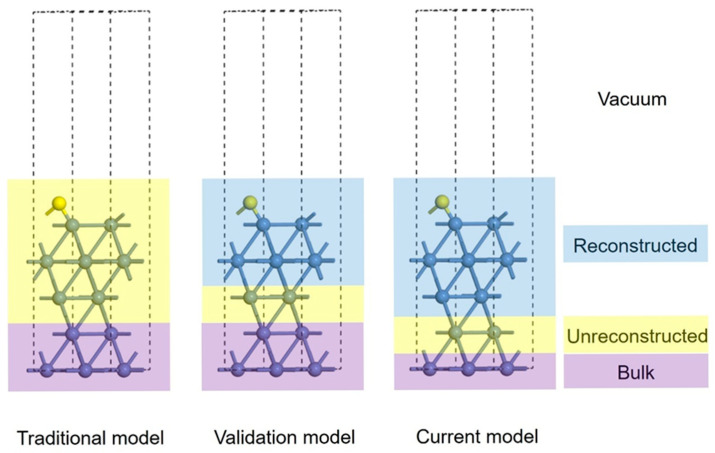
Illustration of the five-layer slabs of the ($\sqrt{3}\times \sqrt{3}$) Ni(111) surface, representing the traditional, validation, and current models. Blue and yellow balls represent Ni and S atoms, respectively. Four different regions are marked, including the vacuum, reconstructed, unreconstructed, and bulk regions.

To simulate the surface structures of Ni sulfides, five-layer slab models of low-Miller-index Ni(111) surfaces were built, as shown in Figure 1, including the traditional model, the validation model, and the current model, respectively. For the traditional model, which is optimized by the DFT method alone, the bottom two layers were fixed and treated as the bulk region, while the top three layers were allowed to relax, comprising the unreconstructed region. For the validation model, which is used to prove the advantage in structure searching by the DFT-based PSO methods, the bottom two layers were also fixed and treated as the bulk region, while the top two layers were deposited along with the surface S atoms, regarded as the reconstructed region. The middle layer was allowed to relax, which is the unreconstructed region. By the DFT-based PSO methods, the current model was applied to determine the structures of sulfide phases on Ni(111) surfaces. In this model, the bottom layer was fixed and treated as the bulk region, while the top three layers were deposited along with the surface S atoms, regarded as the reconstructed region. The middle layer (the second-to-bottom layer) was allowed to relax, comprising the unreconstructed region. The term “relax” means that the full geometry optimization was performed without symmetry restriction. The five-layer Ni(111) slab was constructed with a ($\sqrt{3}\times \sqrt{3}$) surface supercell, and each Ni layer contained three Ni atoms. The corresponding model size was 4.316 Å × 4.316 Å × 23.069 Å. The Brillouin region was uniformly sampled with a grid of 5 × 5 × 1 *k* points. The *k* point testing with a 9 × 9 × 1 grid was conducted on the pristine Ni(111) surface and three saturated nickel sulfide surfaces. The observed energy differences between 9 × 9 × 1 and 5 × 5 × 1 grids were below 0.1 eV in all cases (see Table S2 of the Supplementary Materials), demonstrating that the adopted 5 × 5 × 1 grid sufficiently maintained calculation accuracy while remaining computationally feasible. To evaluate the degree of sulfurization on Ni surfaces, the sulfur coverage (*θ*_S_) is defined as followed:

$$\;\theta_{S}=\frac{N_{S}}{N_{Ni}}$$

where *N*_S_ represents the number of deposited S atoms, and *N*_Ni_ denotes the number of top-surface Ni atoms for each slab model. For ($\sqrt{3}\times \sqrt{3}$) Ni(111), *N*_Ni_ is 3.

## 3. Results

**Figure 2:**
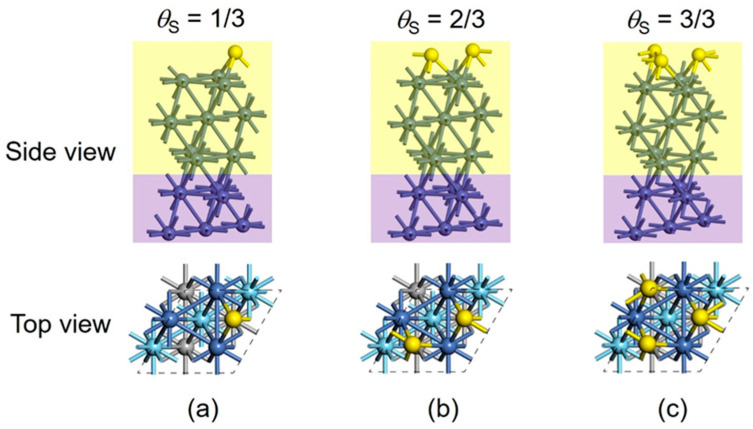
(**a**–**c**) Traditional models of a five-layer Ni(111) slab with a ($\sqrt{3}\times \sqrt{3}$) supercell for S adsorption and stepwise sulfurization. The full geometry optimizations were performed for both the surface S atoms and the top three layers, while the bottom two-layer Ni atoms were fixed in the bulk positions. In the side view, the unreconstructed and bulk regions are marked as yellow and purple, respectively. In the top view, blue, cyan, and gray balls represent the first-, second-, and third-layer Ni atoms, respectively, while the Ni atoms in the bulk region are omitted for simplicity.

As shown in Figure 2, we begin with the classic DFT study of surface S adsorption on Ni(111). The five-layer Ni(111) slab with a ($\sqrt{3}\times \sqrt{3}$) supercell was built via a “traditional” approach, in which the full geometry optimizations were performed for both the surface S atoms and the uppermost three layers, without symmetry restriction, while the bottom two-layer Ni atoms were fixed in the positions at the calculated bulk lattice constant. Our DFT calculations suggest that the S atoms prefer to adsorb at the hollow sites, in agreement with previous DFT results [7,47,48]. *θ*_S_ equals 1/3 when one S atom adsorbs at a hollow site, and increases to 1 when three S atoms are present simultaneously. Further addition of S atoms to the surface causes non-convergence in structural optimization calculations. The internal reason for non-convergence is the unstable layered sulfur configuration formed by excessive S atom deposition on Ni(111). Through the traditional modeling and optimization approach, no reconstruction of the Ni layer was observed for S adsorption on Ni(111), and the deposited S atoms are only detained on the surface. This result might not represent surface metal sulfide phases, especially when high S coverages are involved.

**Figure 3:**
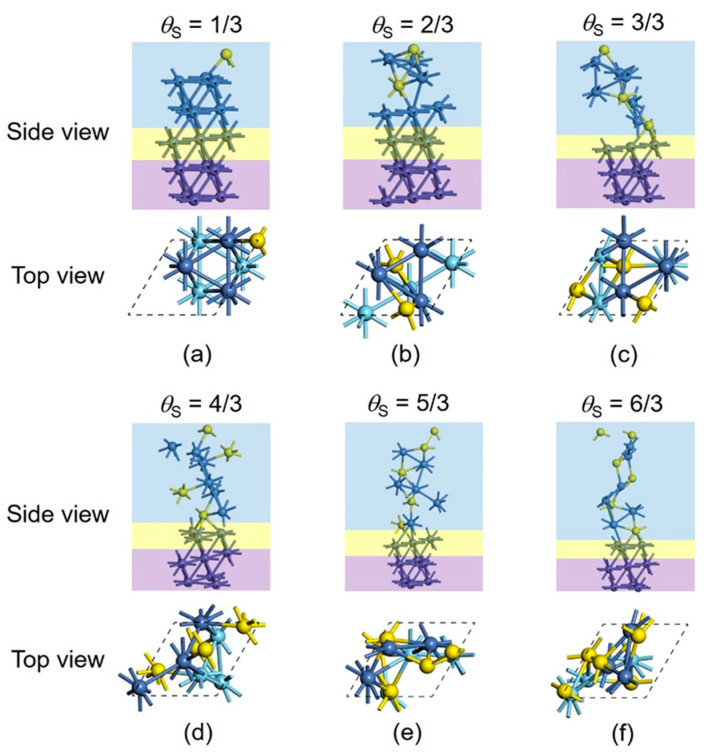
(**a**–**f**) Validation models of a five-layer Ni(111) slab with a ($\sqrt{3}\times \sqrt{3}$) supercell for S adsorption and stepwise sulfurization. The top two Ni layers (six Ni atoms) along with the S atoms are defined as the reconstructed region, while the bottom two Ni layers were fixed as the bulk region, and the middle Ni layer was allowed to relax as the unreconstructed region. In the side view, the reconstructed, unreconstructed, and bulk regions are marked as blue, yellow, and purple, respectively. In the top view, blue and cyan balls represent the first- and second-layer Ni atoms, respectively, while the Ni atoms in the unreconstructed and bulk regions are omitted for simplicity.

In contrast with the above traditional case, significant geometric changes are sampled for the S adsorption when using the PSO method. We constructed another five-layer Ni(111) slab with a ($\sqrt{3}\times \sqrt{3}$) supercell, denoted as the validation model (Figure 3). The bottom two Ni layers were fixed as the bulk region and the third layer was allowed to relax as the unreconstructed region, while the top two Ni layers (six Ni atoms), along with the S atoms, were defined as the reconstructed region. When one S atom adsorbs (*θ*_S_ = 1/3), the most stable structure obtained via the PSO method is almost the same as the above case for the traditional approach. The differences of relevant bond lengths (Ni–Ni and Ni–S) between the two model systems (Figure 2a vs. Figure 3a) are less than 0.01 Å, and the difference in the total energies is only 0.018 eV. When two S atoms are considered (*θ*_S_ = 2/3), the top Ni layer undergoes a substantial reconstruction due to the S adsorption (Figure 3b), and the three Ni atoms of the subsurface layer remain at the bulk lattice site. The new-formed surface sulfide phase in the top layer can be regarded as Ni_3_S_2_. The total energy of the PSO-optimized slab model (Figure 3b) is 0.746 eV lower than the slab model (Figure 2b) optimized via the traditional method, demonstrating the advantage of the PSO method in searching for the thermodynamically stable structure. For the adsorption of three S atoms, the top two Ni layers (deposited six Ni atoms) are both involved in the reconstruction. The new-formed surface sulfide phase is Ni_6_S_3_, and can be denoted as Ni_2_S for simplicity. This reconstructed slab (Figure 3c) is substantially more stable than the unreconstructed one (Figure 2c), with the difference in the total energies increased to 3.430 eV. Notice that, for *θ*_S_ = 1/3 and 2/3, the surface sulfurization is only limited to the top Ni layer, while the subsurface Ni layer is not affected, but the third S atom penetrates the subsurface Ni layer when *θ*_S_ reaches 1, which causes the reconstruction and sulfurization of this layer. As shown in Figure 3d–f, the further addition of S atoms to four, five, and six, corresponding to the *θ*_S_ of 4/3, 5/3 and 6/3, also leads to the reconstruction of the top two Ni layers, whilst the third layer remains almost unchanged because the PSO method is not applied in the unreconstructed region.

**Figure 4:**
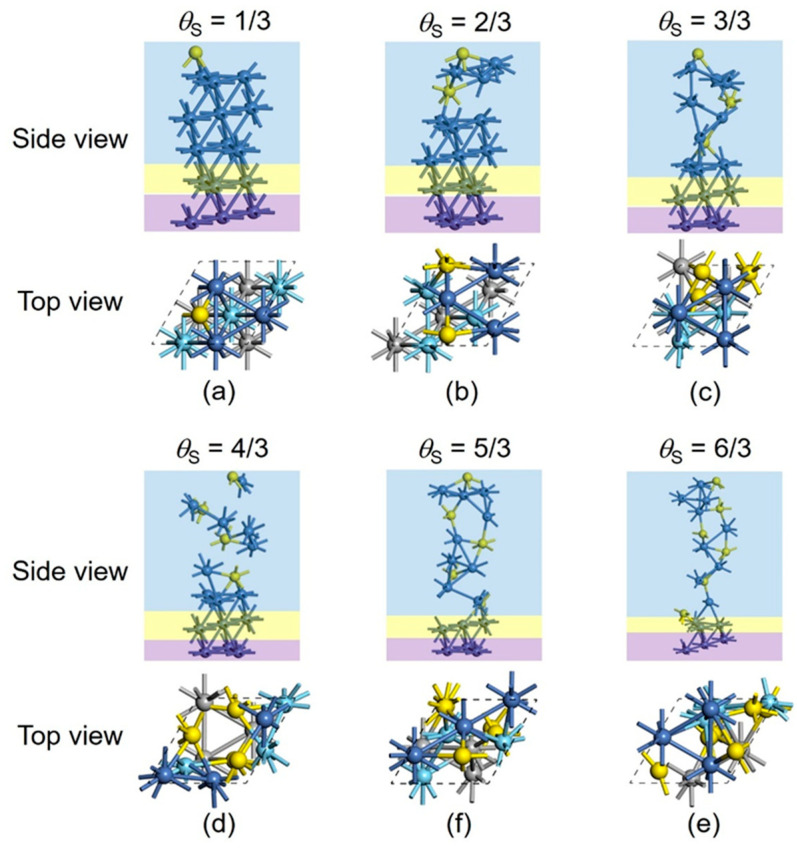
(**a**–**f**) Current models of five-layer Ni(111) slab with a ($\sqrt{3}\times \sqrt{3}$) supercell for stepwise sulfurization. The top three Ni layers, along with the S atoms, are the reconstructed region, the bottom layer is fixed as the bulk region, and the second layer from the bottom is allowed to relax as the unreconstructed region. In the side view, the reconstructed, unreconstructed, and bulk regions are marked as blue, yellow, and purple, respectively. In the top view, blue, cyan, and gray balls represent the first-, second-, and third-layer Ni atoms, respectively, while the Ni atoms in the unreconstructed and bulk regions are omitted for simplicity.

The possibility of the Ni sulfide formation in a deeper Ni layer, such as the third layer from the top, was further investigated and confirmed through the current ($\sqrt{3}\times \sqrt{3}$) Ni(111) slab mentioned in Section 2 (Figure 1) via the PSO method. This model also contains five Ni layers, among which the top three layers (nine Ni atoms in total), along with the S atoms, were deposited in the model system as the reconstructed region, the bottom layer was fixed as the bulk region, and the second layer from the bottom was allowed to relax as the unreconstructed region. For *θ*_S_ from 1/3 to 4/3 (Figure 4a–d), structural and energetic variations are small compared with the cases (Figure 3a–d) where only the top two layers are involved in the reconstruction. The energy differences between systems are less than 0.025 eV. For models with *θ*_S_ of 5/3 (Figure 4e) and 6/3 (Figure 4f), the addition of the fifth and sixth S atoms, leads to the sulfurization of the third Ni layer from the top, and the corresponding reconstruction accounts for a drop in total energies by ~0.4 eV compared with the cases (Figure 3e,f) that only the top two layers are included in the reconstruction. This result confirms that the PSO method can investigate a greater range of structures compared to the “traditional” approach. It can be concluded that each pair of S atoms will at most sulfurize three Ni atoms, which is one layer in our models, and extra S atoms will further sulfurize the next Ni layer to reach saturation. For the current ($\sqrt{3}\times \sqrt{3}$) Ni(111) slab models, the saturated Ni sulfide phase is Ni_3_S_2_ (Figure 4b,d,f), while the unsaturated phases are the intermediate states, such as Ni_3_S (Figure 4a), Ni_2_S (Figure 4c), and Ni_9_S_5_ (Figure 4e). Table 1 lists the structural parameters within the sulfurized layers of current models, including the average bond lengths (*d*) of Ni–S and Ni–Ni bonds and the average bond angle (∠Ni-S-Ni). With the addition of S atoms and the formation of saturated sulfurization state (Ni_3_S_2_) in a deeper Ni layer, the structural parameters of surface-formed Ni sulfides exhibit progressive convergence toward the Ni_3_S_2_ bulk. The fully sulfurized surface exhibits structural characteristics analogous to the Ni_3_S_2_ bulk [58,59].

Based on the current ($\sqrt{3}\times \sqrt{3}$) Ni(111) slab model (Figure 1) and a series of sulfurized states (Figure 4), ab initio thermodynamics was considered to determine the most stable sulfide structures of the Ni(111) surface as a function of temperature (*T*) and partial pressures of H_2_ ($P_{H_{2}}$) and H_2_S ($P_{H_{2}S}$). The surface stability is evaluated by the Gibbs surface energy (*G*_surface_), which can be defined as follows:

$$\;G_{surface}=\frac{G_{total}-nG_{ref}-m\mu_{s}}{2A}$$

where *G*_total_ and *G*_ref_ are the free energies of the sulfurized Ni(111) surface and a single bulk Ni atom, respectively. The chemical potential of S, *μ_s_*, is the total energy of S in the structure. The *n* and *m* represent the number of Ni and S atoms, respectively. *A* is the area of the surface. The *µ_s_* is the chemical potential of sulfur in the gas phase and can be calculated by the following equation:

$$\;\mu_{s}=\mu_{H_{2}S}-\mu_{H_{2}}$$

**Figure 5:**
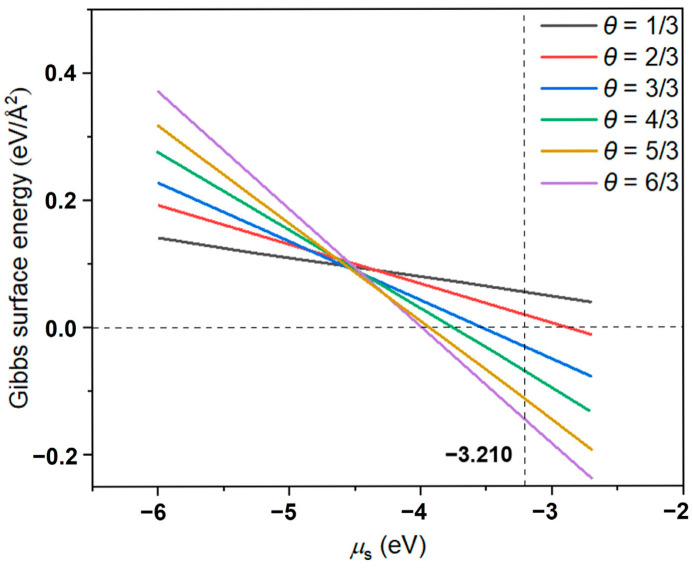
The phase diagram of Gibbs surface energy as a function of chemical potential of sulfur (*μ*_s_) for the sulfurized Ni(111) surfaces with different S coverage (*θ*_S_). The typical RADS reaction condition is *T* = 673 K and $P_{H_{2}}$= 1 MPa, corresponding to *μ*_s_ of −3.210 eV.

The chemical potentials of the gas-phase mixture (H_2_ + H_2_S) are evaluated using the general thermodynamic formula as follows:

$$\;\mu_{s}(T)=\Delta E_{elec}+\Delta ZPE+\Delta H(T)-T\Delta S+RTln\left(\frac{p_{H_{2}S}}{p_{H_{2}}}\right)$$

where ∆*H*(*T*) and ∆*S*(*T*) are the enthalpy and entropy differences, respectively, and ∆*E*_elec_ is the internal energy difference (*E*_elec_(H_2_S) − *E*_elec_(H_2_)), which is approximated by their zero-temperature DFT total energies. The translational and rotational parts are included in the ∆*S*(*T*) terms. Since the S atoms are incorporated into the surface lattice framework during sulfurization process, the entropy term can be negligible when calculating the Gibbs surface energy [60]. ∆*ZPE* represents the zero-point vibrational energy term. The RADS reaction includes thiophene desulfurization on the active center Ni, the hydrogenation of removed S to H_2_S, and the H_2_S desorption from Ni followed by H_2_S adsorption by ZnO support. Under typical reaction conditions (Ni + H_2_S ⇔ NiS_x_ + H_2_), $\frac{p_{H_{2}S}}{p_{H_{2}}}$ is 0.05 [28]. As shown in Figure 5, we obtained four stable sulfurized surfaces, in which the *θ*_S_ are 1/3, 3/3, 5/3, and 6/3, respectively. When *μ*_s_ is less than −4.592 eV, the most stable surface is with *θ*_S_ of 1/3, and the corresponding sulfide phase is Ni_3_S. For a small chemical potential interval of −4.592 to −4.548 eV, the most stable surface is with *θ*_S_ of 3/3, and the corresponding sulfide phase is Ni_2_S. When *μ*_s_ = −4.548 ~ −4.232 eV, the sulfide layer with *θ* = 5/3 becomes the most thermodynamically stable, and the surface sulfide is Ni_9_S_5_. The further increase in the sulfurization potential (*μ*_s_ > −4.232 eV) means the most stable phase evolves into Ni_3_S_2_, in which all top-three Ni layers are engaged in sulfurization and reach the saturated sulfurization state. Thus, under typical RADS reaction conditions of *T* = 673 K and $P_{H_{2}}$= 1 MPa (*μ*_s_ = −3.210 eV), the corresponding sulfide phase is Ni_3_S_2_, which is in good agreement with the sulfur K-edge XANES observations [26].

**Figure 6:**
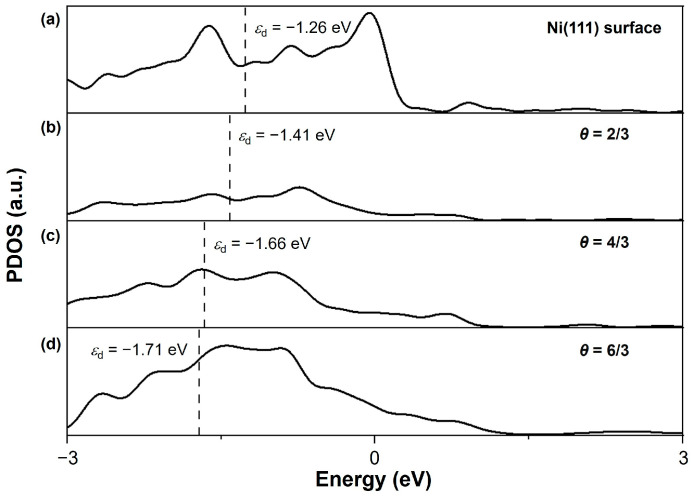
The partial density of states (PDOS) for (**a**) pristine Ni(111) and (**b**–**d**) three saturated nickel sulfide surfaces (*θ*_S_ = 2/3, 4/3, 6/3), along with the values of the *d*-band center (*ε*_d_). The zero energy corresponds to the Fermi level.

The partial density of states (PDOS) for pristine Ni(111) and three saturated nickel sulfide surfaces (*θ*_S_ = 2/3, 4/3, 6/3) are shown in Figure 6. A direct correlation between Ni–S bond strength and the *d*-band center (*ε*_d_) position relative to the Fermi level can be revealed: Progressive stabilization occurs as *ε*_d_ shifts away from the Fermi level (pristine Ni: −1.26 eV → *θ*_S_ = 6/3 sulfide: −1.71 eV). This trend confirms the stability of the *θ*_S_ = 6/3 configuration (*ε*_d_ = −1.71 eV), consistent with previous phase diagram observations. In addition, the interface between sulfurized and pristine Ni layers involves chemical bonding rather than physical adsorption, analogous to oxide layer formation on metals. At this interface (see Table S3 of the Supplementary Materials), Ni-Ni bonds exhibit weakened interactions (average 2.692 Å vs. bulk 2.490 Å), while Ni-S bonds demonstrate enhanced strength (average 2.203 Å vs. Ni_3_S_2_ bulk 2.336 Å).

## 4. Conclusions

Density functional theory-based particle swarm optimization methods were used to explore the low-energy structures of a sulfide phase on the Ni(111) surface and the corresponding formation mechanism. Traditional modeling and optimization approaches via DFT were incapable of exploring the sulfurized structures formed by the Ni(111) surface, especially at high S coverages. We confirmed that the PSO method is able to search for the thermodynamically more stable structures. Based on the ($\sqrt{3}\times \sqrt{3}$) Ni(111) slab models, a sulfurization limit of surface sulfide phases was identified. Each two deposited S atoms will at most sulfurize a Ni layer containing three Ni atoms (Ni:S = 3:2), and extra S atoms sulfurize the next Ni layer until saturation. Under the typical RADS reaction condition of *T* = 673 K and $P_{H_{2}}$= 1 MPa (*μ*_s_ = −3.210 eV), the corresponding sulfide phase is determined to be Ni_3_S_2_ by ab initio thermodynamics, which is in good agreement with the sulfur K-edge XANES observations. The saturated Ni sulfide phase is Ni_3_S_2_, while unsaturated intermediate states, such as Ni_3_S, Ni_2_S, and Ni_9_S_5_, are observed. These stable unsaturated sulfide structures might explain the existence of the various Ni sulfide phases with different ratios of Ni to S observed in previous experiments.

## Figures and Tables

**Table 1:** Average bond lengths (*d*) of Ni–S and Ni–Ni bonds and average bond angle (∠Ni-S-Ni) of sulfide phases in current models.

*θ*_S_	*d*_Ni-S_ (Å)	*d*_Ni-Ni_ (Å)	∠Ni-S-Ni
1/3	2.137	2.478	72.2
2/3	2.191	2.529	71.4
3/3	2.208	2.586	71.8
4/3	2.232	2.605	72.4
5/3	2.261	2.609	71.1
6/3	2.294	2.595	69.2
Ni_3_S_2_ bulk [58,59]	2.336	2.585	69.1

## Data Availability

The data are contained within the article.

## Supplementary Materials

The following supporting information can be downloaded at https://www.mdpi.com/article/10.3390/nano15110788/s1. The detailed PSO protocol; Figures S1–S4. Lowest-energy structures of sulfurized surfaces (*θ*_S_ = 1/3 − 6/3) in validation models and current models; Table S1. Energetic discrepancies between pre- and post-dipole correction states for the current models; Table S2. The *k* point testing and energy comparisons with a 9 × 9 × 1 grid conducted on the pristine Ni(111) surface and three saturated nickel sulfide surfaces of the current models; Table S3. Relevant bond lengths within the interface between sulfurized and underneath pristine Ni layers.

## Abbreviations

The following abbreviations are used in this manuscript:

DFT	Density functional theory
PSO	Particle swarm optimization
Ni	Nickel
XANES	X-ray absorption near-edge structure
HDS	Hydrodesulfurization
RADS	Reactive adsorption desulfurization
XRD	X-ray diffraction
Ni_x_S_y_ or NiS_x_	Ni sulfides
CALYPSO	Crystal structure AnaLYsis by Particle Swarm Optimization
VASP	Vienna ab initio simulation package
PAW	Projector augmented wave method
GGA-PBE	Generalized gradient approximation developed by Perdew–Burke–Ernzerhof
