# Supplementary material for: A Density Functional Theory-Based Particle Swarm Optimization Investigation of Metal Sulfide Phases for Ni-Based Catalysts
Source: Nanomaterials (Basel). 2025 May 23;15(11):788. doi: 10.3390/nano15110788 (PMC12156416; doi:10.3390/nano15110788)
Supplement: Supplementary file 1 [file nanomaterials-15-00788-s001.zip › nanomaterials-3637377-supplementary.pdf]

Supporting Information

A Density Functional Theory-Based Particle Swarm Optimization Investigation of Metal Sulfide Phases for Ni-Based Catalysts

Houyu Zhu [1,*], Xiaohan Li [1], Xiaoxin Zhang [2], Yucheng Fan [1], Xin Wang [1], Dongyuan Liu [1], Zhennan Liu [1], Xiaoxiao Gong [2,*], Wenyue Guo [1] and Hao Ren [1,*]

[1] Shandong Key Laboratory of Intelligent Energy Materials, School of Materials Science and Engineering, China University of Petroleum (East China), Qingdao 266580, China; z23140070@s.upc.edu.cn (X.L.); z22140074@s.upc.edu.cn (Y.F.); z23140055@s.upc.edu.cn (X.W.); b21140013@s.upc.edu.cn (D.L.); z23140098@s.upc.edu.cn (Z.L.); wyguo@upc.edu.cn (W.G.)

[2] State Key Laboratory of Petroleum Molecular & Process Engineering, SINOPEC Research Institute of Petroleum Processing Co., Ltd., Beijing 10083, China; zhangxx.ripp@sinopec.com

* Correspondence: hyzhu@upc.edu.cn (H.Z.); gongxiaoxiao.ripp@sinopec.com (X.G.); renh@upc.edu.cn (H.R.)

The Particle Swarm Optimization (PSO) protocol integrated within the CALYPSO framework is designed to predict low-energy surface configurations through a combination of global exploration and local refinement. Each particle in the swarm represents a candidate surface structure, and its trajectory in the search space is guided by both its historical best position (p_{best}) and the global best position (g_{best}) identified by the swarm. The algorithm iteratively updates particle velocities and positions to minimize the total energy (E_{DFT}) of the system. Initialization begins with a substrate model defined by lattice vectors $a = 4.316$ Å, $b = 3.738$ Å, $c = 30.069$ Å, and different quantities of Ni and S atoms are delivered. Surface optimization is constrained to a 9 Å thickness layer, with a forbidden thickness of 0.5 Å applied to bulk atoms.

The PSO parameters include a population size of 30 and a maximum of 20 generations. The inertia weight (w) decreases linearly from 0.9 to 0.4 over iterations, while acceleration constants c_1 and c_2 are set to 2.0. Random numbers r_1 and r_2, uniformly distributed in [0,1], introduce stochasticity. Velocity and position updates follow:

$$v_i^{t+1} = wv_i^t + c_1 r_1 (p_{best,i}^t - x_i^t) + c_2 r_2 (g_{best,i}^t - x_i^t)$$

$$x_i^{t+1} = x_i^t + v_i^{t+1}$$

where v_i^t is the velocity of particle i at iteration t, x_i^t is its position, $p_{best,i}^t$ is its historical best position, and $g_{best,i}^t$ is the global best position.

Fitness evaluation involves DFT relaxations using VASP with parameters: *ISMEAR* = 0, *SIGMA* = 0.05, *EDIFF* = 3×10^{-4} eV, and *EDIFFG* = −0.3 eV/Å. The total

energy E_{DFT} extracted from OUTCAR serves as the fitness value. Termination criteria include energy convergence (< 1 meV/atom variation over 5 generations) or reaching the maximum generation limit. Post-processing ranks structures by E_{DFT}, selects the top 5 for phonon stability analysis using Phonopy and compares predicted lattice constants (*a* = 4.316 Å, *b* = 3.738 Å,) with experimental data.

Table S1. Energetic discrepancies (ΔE, eV) between pre- and post-dipole correction states for the current models.

θ_S	E_{pre}	E_{post}	ΔE
1/3	-84.171675	-84.171300	0.000375
2/3	-88.525613	-88.525631	-0.000018
3/3	-93.378396	-93.379133	-0.000737
4/3	-97.787520	-97.787070	0.000450
5/3	-101.915039	-101.906521	0.008518
6/3	-106.406844	-106.406583	0.000261

Table S2. The *k* point testing and energy comparisons (ΔE, eV) with a 9×9×1 grid conducted on the pristine Ni(111) surface and three saturated nickel sulfide surfaces of the current models.

θ_S	$E_{5\times5\times1}$	$E_{9\times9\times1}$	ΔE
0	-78.181	-78.155	0.026
2/3	-88.515	-88.511	0.004
4/3	-97.799	-97.801	-0.002
6/3	-106.681	-106.649	0.032

Table S3. Relevant bond lengths (d_{Ni-Ni} and d_{Ni-S}, Å) within the interface between sulfurized and underneath pristine Ni layers.

θ_S	d_{Ni-S}	d_{Ni-Ni}
2/3	2.210	
4/3	2.167	2.523
	2.207	2.793
6/3	2.210	2.600
	2.223	2.851
Ni bulk		2.490
Ni_3S_2 bulk[58, 59]	2.336	

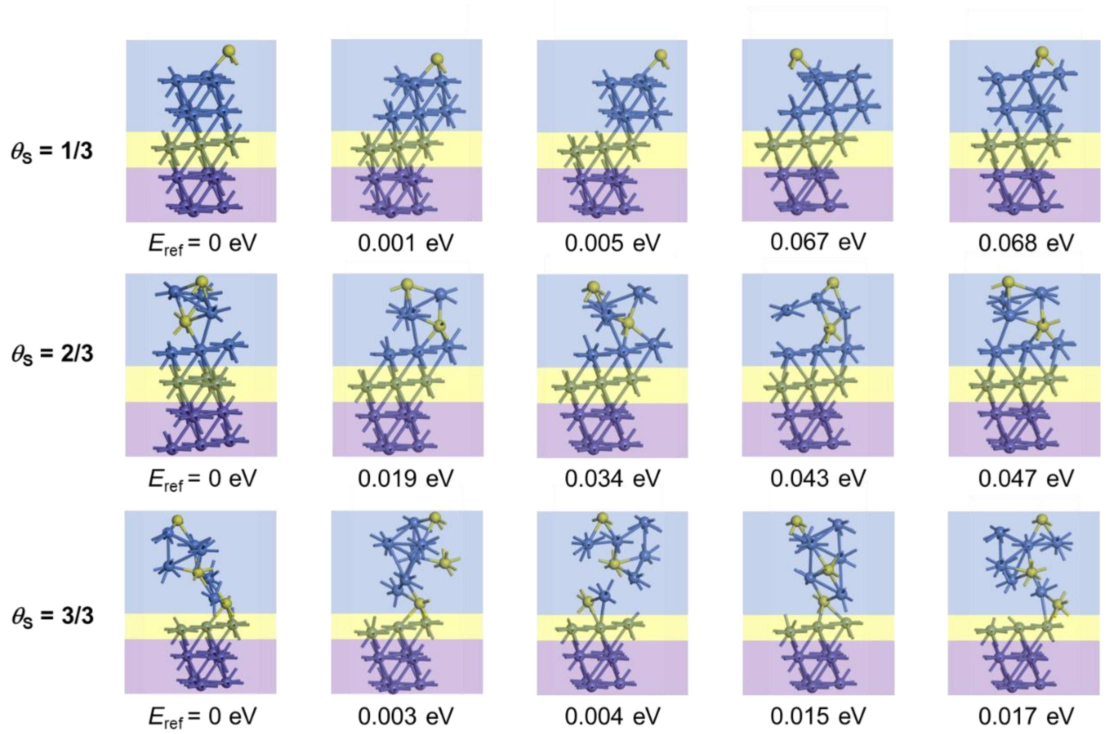

Figure S1. Five lowest-energy structures of sulfurized surfaces (θ_S = 1/3 – 3/3) in Validation models. The most stable ones are chosen to represent the global minimum geometries for each sulfurized surface, and the corresponding total energies are taken as the energy reference (E_{ref}, eV).

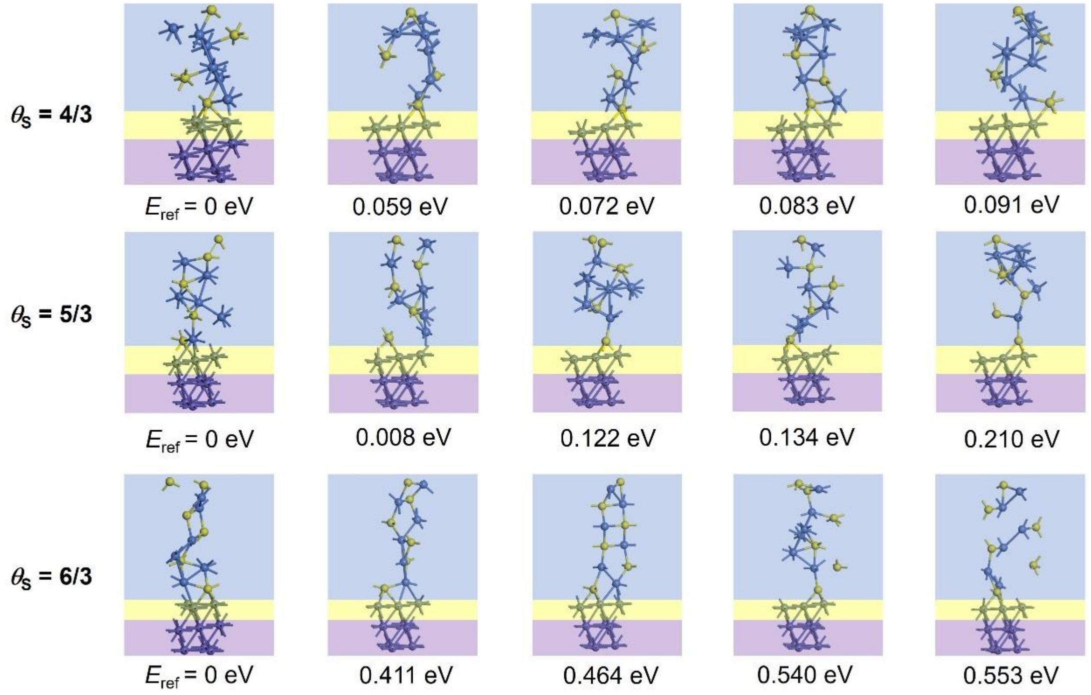

Figure S2. Five lowest-energy structures of sulfurized surfaces (θ_S = 4/3 – 6/3) in Validation models. The most stable ones are chosen to represent the global minimum geometries for each sulfurized surface, and the corresponding total energies are taken as the energy reference (E_{ref}, eV).

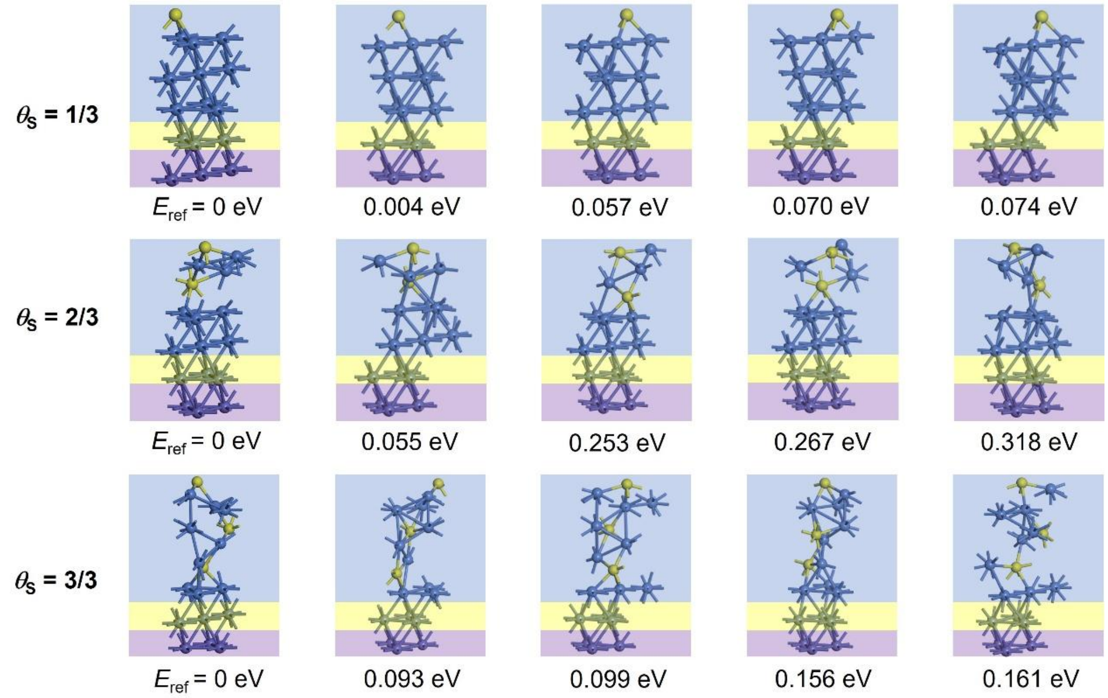

Figure S3. Five lowest-energy structures of sulfurized surfaces (θ_S = 1/3 – 3/3) in Current models. The most stable ones are chosen to represent the global minimum geometries for each sulfurized surface, and the corresponding total energies are taken as the energy reference (E_{ref}, eV).

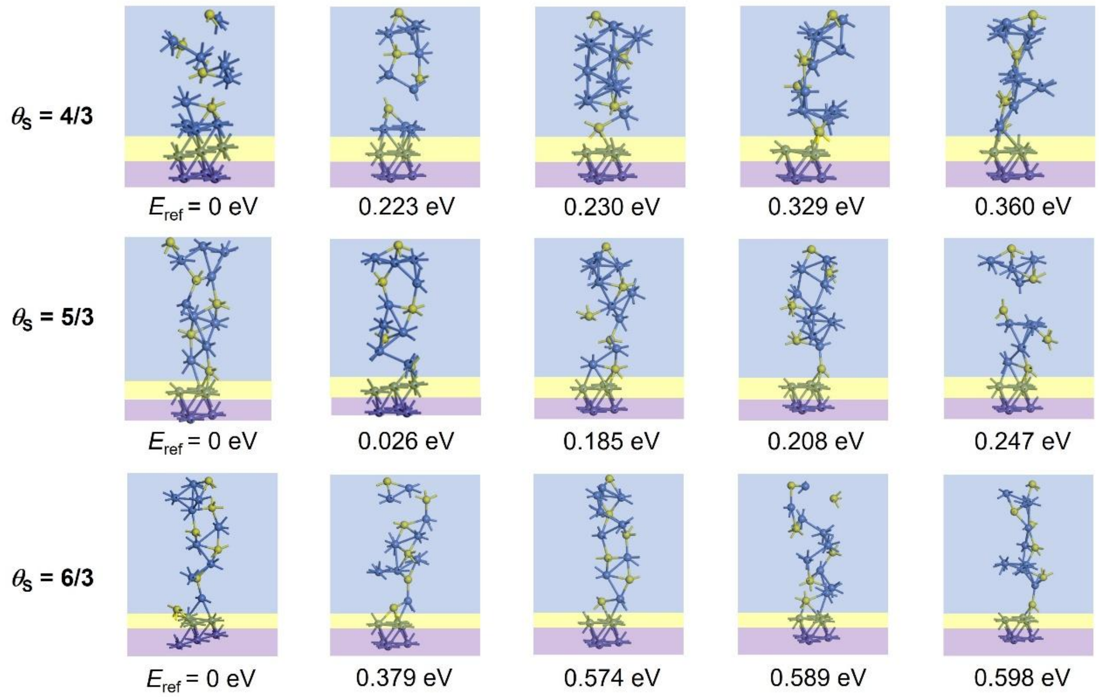

Figure S4. Five lowest-energy structures of sulfurized surfaces (θ_S = 4/3 – 6/3) in Current models. The most stable ones are chosen to represent the global minimum geometries for each sulfurized surface, and the corresponding total energies are taken as the energy reference (E_{ref}, eV).
